# Interaction of Alpha Synuclein and Microtubule Organization Is Linked to Impaired Neuritic Integrity in Parkinson’s Patient-Derived Neuronal Cells

**DOI:** 10.3390/ijms23031812

**Published:** 2022-02-05

**Authors:** Lukas Seebauer, Yanni Schneider, Alice Drobny, Sonja Plötz, Tomas Koudelka, Andreas Tholey, Iryna Prots, Beate Winner, Friederike Zunke, Jürgen Winkler, Wei Xiang

**Affiliations:** 1 Department of Molecular Neurology, University Hospital Erlangen, Friedrich-Alexander Universität, Erlangen-Nürnberg (FAU), 91054 Erlangen, Germany; lukas-seebauer@live.de (L.S.); Yanni.Schneider@uk-erlangen.de (Y.S.); Alice.Drobny@uk-erlangen.de (A.D.); Sonja.Ploetz@uk-erlangen.de (S.P.); friederike.zunke@fau.de (F.Z.); Juergen.Winkler@uk-erlangen.de (J.W.); 2 Systematic Proteome Research & Bioanalytics, Institute for Experimental Medicine, Christian-Albrechts-Universität zu Kiel, 24105 Kiel, Germany; t.koudelka@iem.uni-kiel.de (T.K.); a.tholey@iem.uni-kiel.de (A.T.); 3 Department of Stem Cell Biology, University Hospital Erlangen, Friedrich-Alexander Universität Erlangen-Nürnberg (FAU), 91054 Erlangen, Germany; iryna.prots@uk-erlangen.de (I.P.); beate.winner@fau.de (B.W.); 4 Center of Rare Diseases Erlangen (ZSEER), University Hospital Erlangen, Friedrich-Alexander Universität Erlangen-Nürnberg (FAU), 91054 Erlangen, Germany

**Keywords:** alpha-synuclein, *SNCA* duplication, Parkinson’s disease, microtubule, neurite, iPSC, neurodegeneration

## Abstract

Parkinson’s disease (PD) is neuropathologically characterized by the loss of dopaminergic neurons and the deposition of aggregated alpha synuclein (aSyn). Mounting evidence suggests that neuritic degeneration precedes neuronal loss in PD. A possible underlying mechanism could be the interference of aSyn with microtubule organization in the neuritic development, as implied by several studies using cell-free model systems. In this study, we investigate the impact of aSyn on microtubule organization in aSyn overexpressing H4 neuroglioma cells and midbrain dopaminergic neuronal cells (mDANs) generated from PD patient-derived human induced pluripotent stem cells (hiPSCs) carrying an aSyn gene duplication (*SNCA^Dupl^*). An unbiased mass spectrometric analysis reveals a preferential binding of aggregated aSyn conformers to a number of microtubule elements. We confirm the interaction of aSyn with beta tubulin III in H4 and hiPSC-derived mDAN cell model systems, and demonstrate a remarkable redistribution of tubulin isoforms from the soluble to insoluble fraction, accompanied by a significantly increased insoluble aSyn level. Concordantly, *SNCA^Dupl^* mDANs show impaired neuritic phenotypes characterized by perturbations in neurite initiation and outgrowth. In summary, our findings suggest a mechanistic pathway, through which aSyn aggregation interferes with microtubule organization and induces neurite impairments.

## 1. Introduction

Parkinson’s disease (PD) is the most common neurodegenerative movement disorder worldwide, clinically hallmarked by motor symptoms, such as bradykinesia, rigidity, and resting tremor. Neuropathologically, PD is characterized by the progressive loss of dopaminergic neurons in the substantia nigra pars compacta (SNpc) of the midbrain and the deposition of intracellular inclusions, referred to as Lewy bodies and Lewy neurites within neuronal cell bodies and processes, respectively [1]. The characterization of Lewy pathology revealed that the protein alpha synuclein (aSyn), in conjunction with other deposited proteins, lipids and organelles, is one of the main components of Lewy inclusions [2,3].

aSyn is a small intracellular protein with a molecular weight of 14 kDa. The protein is abundantly expressed in neurons and localized in the cytoplasm, presynaptic terminals, and nucleus [4]. Initial genetic evidence unequivocally linked aSyn point mutations in the aSyn gene (*SNCA*, former Park 1 and Park 4) or multiplications (duplication or triplication) of the *SNCA* gene locus to monogenic PD [5]. The involvement of aSyn in the pathogenesis of PD was further substantiated by the finding of aSyn deposition in the brain of patients with sporadic PD [3]. Intensive research in recent decades provided the evidence that aSyn exists in aggregated forms in Lewy inclusions, and abnormal aSyn aggregation in conjunction with its deleterious effects plays a pivotal role in both PD pathogenesis and progression.

Given the unique function and shape of neurons, axonal homeostasis and transport are important in maintaining neuronal function and connectivity. Axonal activity is particularly essential for neurons with long-range projections, such as dopaminergic neurons within the nigrostriatal pathway. Mounting evidence suggests that axonal degeneration temporally precedes perikaryon loss and, thus, reflects an early cellular pathological event in PD [6,7]. Moreover, aSyn was shown to be involved in axonal degeneration associated with PD. For example, Koch et al. reported affected neurite morphology and axonal transport in rat primary midbrain neurons overexpressing wild type and mutant aSyn induced by viral transduction [8]. In agreement with these findings in rodent-derived neurons, we observed a severely impaired axonal transport in human cortical projection neurons (CPNs), differentiated from human induced pluripotent stem cells (hiPSC) derived from a PD patient carrying a duplication of the *SNCA* gene locus (*SNCA^Dupl^*) [9]. Moreover, we demonstrated a deleterious effect of aSyn oligomers in triggering axonal transport deficits via the overexpression of oligomer-prone aSyn mutants in hiPSC-neurons. 

The microtubule, a polymer of beta tubulin (bTub) and alpha tubulin (aTub), is one of the core elements of the eukaryotic cytoskeleton [10]. The dynamic assembly of microtubules from heterodimers of aTub and bTub plays the central role in neuritogenesis and neurite function [10]. One possible explanation for aSyn-mediated neuritic deficits, as we observed in PD patient-derived neurons [9], could be the interference of aSyn with microtubule dynamics. Indeed, several studies, mostly based on cell-free experiments, have provided hints about the interaction of aSyn and microtubule dynamics [11,12]. Recently, we characterized hiPSC-derived neurons from *SNCA^Dupl^* patients and compared the phenotypes of *SNCA^Dupl^* carrying midbrain dopaminergic neuronal cells (mDANs) and CPNs with those from control mDANs and CPNs [13]. We showed that increased *SNCA* dosage is linked to elevated levels of aggregated aSyn and reduced viability, specifically in *SNCA^Dupl^* mDANs, recapitulating therefore PD pathology in patient-derived mDANs. Interestingly, we observed a remarkable reduction in beta tubulin III (bTubIII), a neuronal beta tubulin isoform, in *SNCA^Dupl^* mDANs. According to these converging findings, we postulate that aSyn aggregation impacts microtubule dynamics and neurite homeostasis, contributing furthermore to neuritic deficits associated with PD. 

In this study, we made use of aSyn overexpressing H4 neuroglioma cells and neural cell models generated from hiPSCs derived from a PD patient carrying *SNCA^Dupl^* to address the interference of aSyn with the microtubule network. Our results reveal a direct interaction of aSyn, in particular its aggregated forms, with microtubule elements. We further described a link between elevated aSyn levels and aggregation, disturbed tubulin distribution, and impaired neurite morphology. These findings provide an insight into the molecular mechanism, through which degeneration of PD patient-derived neurons might occur.

## 2. Results

### 2.1. aSyn Overexpression Leads to Its Aggregation and Promotes Its Interaction with Microtubule Elements 

**Figure 1:**
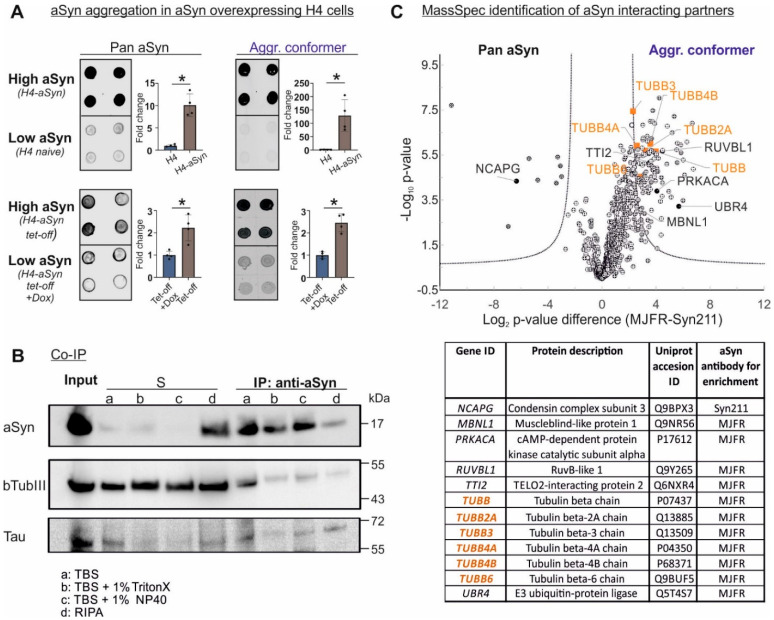
Interaction of aSyn and tubulin in aSyn overexpressing H4 cells. (**A**) Dot blot analysis of total aSyn and aggregated aSyn in aSyn overexpressing H4 cell models (high aSyn: H4-aSyn and H4-aSyn tet-off cells) and their respective low aSyn expressing cell lines (low aSyn, naïve H4 cells and H4-aSyn tet-off+Dox). Total aSyn levels were determined by using a pan aSyn antibody Syn 1, while the levels of aggregated aSyn conformers (aggr. conformer) were assessed by using a conformation-specific antibody MJFR-14-6-4-2. Both aSyn overexpressing cell lines (high aSyn) exhibit higher levels of aSyn aggregates when comparted to their respective low aSyn counterparts. Total protein loaded was controlled by direct blue staining (shown in Supplementary Figure S1). For quantification, the fold change in aSyn level in a high aSyn cell line was calculated by normalization against total protein (direct blue) and the average level in the corresponding low aSyn cells (*n* = 4). Statistics: Mann–Whitney test; * *p* < 0.05. (**B**) Immunoprecipitation of aSyn from H4-aSyn cells using a pan aSyn antibody Syn1. bTubIII and Tau are co-precipitated. For immunoprecipitation, a buffer without detergents (TBS, a), buffers containing non-ionic milder detergents (TBS + 1% Triton X 100, b or TBS + 1% NP40, c), or a buffer containing stronger detergents (RIPA, d) were used. bTubIII and Tau are detectable in all conditions. (**C**) Mass spectrometric identification of proteins co-precipitated with aSyn in H4-aSyn tet-off cells. Co-immunoprecipitation was performed by using the pan aSyn antibody Syn211 or the conformation-specific anti-aSyn antibody MJFR-14-6-4-2. Volcano plots of co-precipitated proteins identified by mass spectrometry are shown (left: proteins co-precipitated with Syn211, right: proteins co-precipitated with MJFR-14-6-4-2). Significant proteins from two independent experiments are over the solid lines. Identified microtubule-associated proteins are highlighted by their gene ID and listed in the table. A two-tailed t-test was performed in Perseus (1.6.10.43) using a permutation-based FDR to account for the multiple testing hypothesis (technical injection replicates were preserved during randomization). Candidates were filtered using an FDR of 1% and a fold change (s0) of 2.

In order to investigate the interference of aSyn, in particular its aggregated forms, with the microtubule network, we employed two different H4 neuroglioma cell lines overexpressing aSyn, H4-aSyn cells and H4-aSyn tet-off cells, and their low aSyn expressing counterparts, naïve H4 cells and H4-aSyn tet-off cells treated with doxycycline (H4-aSyn tet-off+Dox), respectively. Both H4-aSyn and H4-aSyn tet-off cells are characterized by an overall high total aSyn level when compared to naïve H4 and H4-aSyn tet-off+Dox cells, respectively (H4-aSyn vs. naïve H4, fold change: 10.1 ± 2.5, * *p* < 0.05; H4-aSyn tet-off vs. H4-aSyn tet-off+Dox, 2.2 ± 0.6 * *p* < 0.05) (Figure 1A). aSyn overexpression is a well-known risk factor for abnormal aggregation of aSyn, initiated by its conformational changes during the aggregation process. Using non-denaturing dot blot analysis combined with a conformation-specific antibody MJFR-14-6-4-2 with a strong immunoreactivity toward aggregated aSyn [14], we observed a remarkably elevated aSyn aggregation in H4-aSyn cells. In contrast, aggregated conformers were almost not detectable in naïve H4 cells. Concordantly, the high aSyn H4 tet-off cell line also showed a higher level of the aggregated aSyn conformers, which was decreased by doxycycline-dependent aSyn downreguation (H4-aSyn tet-off vs. H4-aSyn tet-off+Dox: 2.4 ± 0.4, * *p* < 0.05). In total, both H4 cell systems revealed a propotional increase in aggregated aSyn linked to aSyn overexpression. 

Previous studies have shown that aSyn is capable of directly binding to microtubules and tubulins [15]. To confirm this interaction, we performed an immunoprecipitation of aSyn from H4-aSyn cells with an aSyn antibody (Syn1), whereby the co-precipitation of bTubIII and aSyn was observed (Figure 1B). The binding affinity was assessed via immunoprecipitation using detergents of different strengths (Figure 1B). The co-precipitation of aSyn and bTubIII was observed, even when using RIPA buffer, containing harsh detergents, indicating a strong binding between aSyn and bTubIII. Furthermore, Tau, a microtubule associated protein, was also co-precipitated with aSyn, further indicating the interaction of aSyn with microtubules.

We next questioned whether aSyn conformers differentially bind microtubule elements. We carried out immunoprecipitation of aSyn by using a pan-aSyn antibody (Syn211), and a conformation-specific antibody (MJFR-14-6-4-2), followed by the liquid chromatography coupled mass spectrometric (LC-MS) analysis of co-precipitated, aSyn interaction proteins. A comparison of total aSyn (Syn211) and aggregated aSyn (MJFR-14-6-4-2) interaction partners identified various bTub isoforms, including bTubIII, as well as several microtubule factors, preferentially interacting with the aggregated aSyn conformers (Figure 1C). The LC-MS analysis substantiated the existence of an aSyn-microtubule interaction. Importantly, it is aggregated aSyn that interacts preferentially with tubulins and other microtubule factors. 

### 2.2. Insoluble aSyn Is Associated with the Redistribution of Microtubule Elements

**Figure 2:**
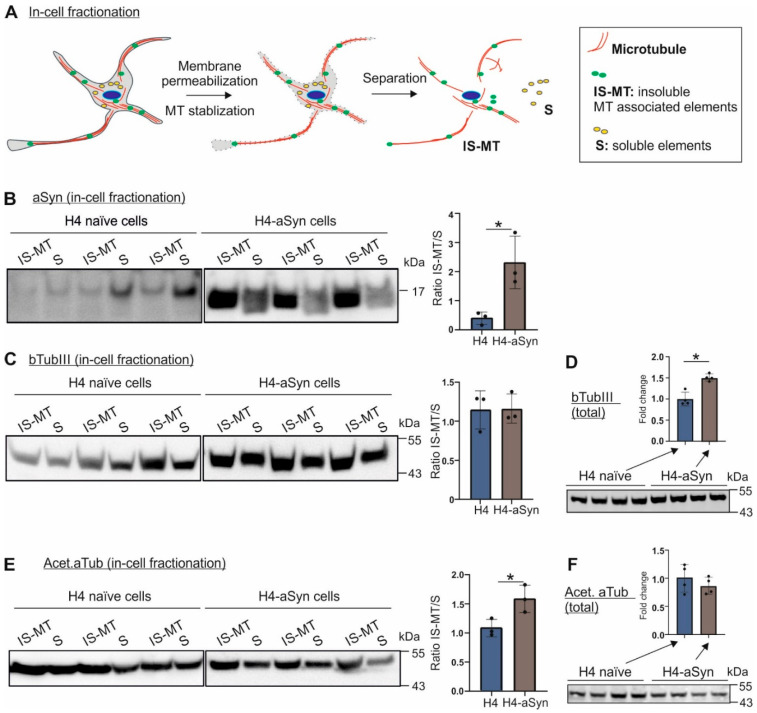
Impact of aSyn overexpression on microtubule organization. (**A**) Scheme of in-cell fractionation approach for separation of soluble (S) and insoluble, microtubule-associated fraction (IS-MT). (**B**,**C**,**E**) WB images (**left**) and quantification (**right**) of aSyn (**B**), bTubIII (**C**), and acetylated aTub (Acet. aTub, E) in S and IS-MT fractions extracted from H4 naïve and H4-aSyn cells. For quantification, the ratio of aSyn, bTubIII or acetylated aTub in the IS-MT versus the S fraction was calculated and values from three experiments were used (*n* = 3). aSyn overexpression in H4 cells leads to a shift of aSyn and acetylated aTub into IS-MT pools. Statistics: unpaired t-test; * *p* < 0.05. (**D**,**F**) WB analysis of total bTubIII (**D**) and acetylated aTub levels (**F**) in H4 naïve and H4-aSyn cells. Fold changes were calculated by normalization against Ponceau intensity (shown in Supplementary Figure S2) and the average level of H4 naïve cells (*n* = 4). The values are shown as mean ± SD. Statistics: Mann–Whitney test; * *p* < 0.05.

Several cell-free studies have provided the hint that aSyn is involved in microtubule nucleation and dynamics [11,12]. We next explored if aSyn overexpression interferes with microtubule organization. We performed an in-cell fractionation approach as described by Katsetos et al. [16], by which insoluble, microtubule-associated elements were enriched and separated from the soluble, microtubule-unbound cell fraction (Figure 2A). Cell fractionation revealed a redistribution of aSyn from soluble fractions to insoluble fractions in H4-aSyn cells compared to naïve H4 cells (Figure 2B), verifying an increased aSyn aggregation under aSyn overexpressing conditions. 

In both naïve H4 and H4-aSyn cells, we detected a substantial amount of bTubIII in the soluble (microtubule-free) and insoluble (microtubule-associated) pools. A redistribution of bTubIII between soluble and insoluble fractions was not clearly detected in aSyn overexpressing H4-aSyn cells (Figure 2C). However, the level of acetylated aTub, which is essential for stabilizing assembled microtubules, in insoluble fractions, was significantly enhanced. Collectively, our results from the H4 cell models suggest that aSyn overexpression induces its aggregation and increases the interaction of its aggregated conformers with microtubule elements and organization. 

We next sought to explore whether the redistribution of acetylated aTub is associated with a changed protein level. We analyzed total protein levels of bTubIII and acetylated aTub by WB analysis (Figure 2D,F). The acetylated aTub levels were unchanged in H4-aSyn compared to naïve H4 cells (Figure 2F). In contrast, bTubIII levels were significantly increased in aSyn overexpressing H4-aSyn cells (Figure 2D). Thus, our result does not support a clear association between the levels of these tubulins and their redistribution. 

### 2.3. SNCA Duplication Leads to an Increased aSyn Protein Level in hiPSC-Derived NPCs and Neuronal Cells

**Figure 3:**
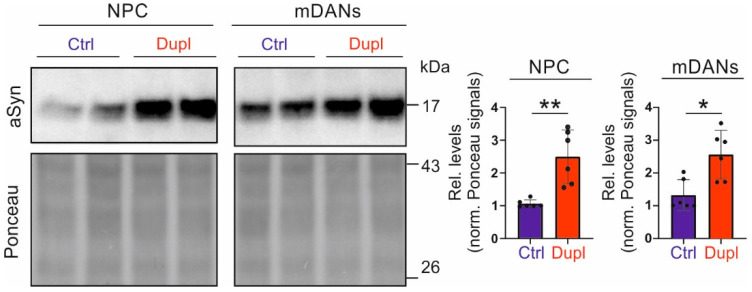
aSyn levels in *SNCA^Dupl^* patient-derived cells. Representative WB images (**left**) and the quantification (**right**) of aSyn levels in NPCs as well as in mDANs differentiated for 10 days, which were generated from healthy controls (Ctrl) and the duplication patient (Dupl), respectively. aSyn levels are significantly higher in *SNCA^Dupl^* NPCs and mDANs compared to control cells. Relative levels were calculated by normalization against Ponceau intensity and the level of a control line in each differentiation round. The values are shown as mean ± SD. Values from two control lines as well as two *SNCA^Dupl^* lines and three independent differentiation rounds per line were used for the quantification (*n* = 3/line). Statistics: Mann–Whitney test; * *p* < 0.05, ** *p* < 0.01.

We next asked whether the observed interaction between aggregated aSyn and microtubule elements and organization in H4 cell models is also relevant to PD. For this, we made use of hiPSC-lines derived from a PD patient with a heterozygous *SNCA^Dupl^*, and compared the phenotypes in *SNCA^Dupl^* neural cells with those in hiPSC-derived cells from two healthy donors. To generate mDANs from hiPSC, we applied a small molecule-based protocol developed by Reinhardt et al. [17] that is well established in our previous studies [13,18,19]. hiPSC differentiation involved the derivation of hiPSCs to the formation of embryoid bodies (EBs), the expansion and differentiation of NPCs, and the maturation of mDANs (Supplementary Figure S3A,B). Immunocytochemical (ICC) analysis revealed expression of the neural progenitor markers nestin and Sox2 in NPCs (Supplementary Figure S3C). In mDANs, the neuron-associated bTub isoform bTubIII, the presynaptic marker synapsin I, and the markers for dopamine synthesis, such as tyrosine hydroxylase (TH) and DOPA decarboxylase (DDC), showed a maturation-dependent increase, as determined either by ICC or Western blot (WB) (Supplementary Figure S3D–G), indicating an expression of characteristic markers for mDANs. In line with our early findings in other *SNCA^Dupl^* hiPSC-derived neuronal cells [9,13], we detected a significantly higher aSyn level in *SNCA^Dupl^* -derived cells, either in NPCs or in mDANs differentiated for 10 days, when compared to control donor-derived cells (Figure 3, NPCs: average 2.3-fold increase, ** *p* < 0.01; mDANs: average 1.9-fold increase, * *p* < 0.05), confirming thereby *SNCA^Dupl^*-mediated aSyn overload in *SNCA^Dupl^* patient-derived neural cells. 

### 2.4. The Level of Tubulins Is Decreased in mDANs Carrying SNCA^Dupl^

**Figure 4:**
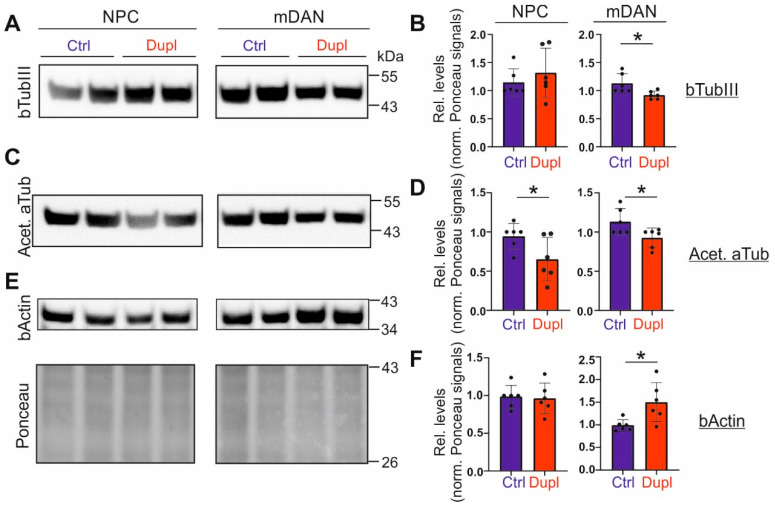
Expression levels of cytoskeletal proteins in *SNCA^Dupl^* patient-derived cells. Representative WB images and the quantification of bTubIII (**A**,**B**), acetylated aTub (acet. aTub) (**C**,**D**) and bActin (**E**,**F**) levels in NPCs as well as mDANs after differentiation for 10 days, generated from healthy controls (Ctrl) and the *SNCA^Dupl^* patient (Dupl), respectively. bTubIII and acetylated aTub levels are significantly reduced in mDANs carrying *SNCA^Dupl^* as compared to control mDANs from healthy individuals, while bActin levels are significantly increased in *SNCA^Dupl^* mDANs. Relative levels were calculated by normalization against Ponceau intensity and the level of a control line in each differentiation round. The protein loading control with Ponceau staining for bTubIII is shown in this figure. Ponceau staining for acetylated aTub and bActin is provided in Supplementary Figure S4A. The values are shown as mean ± SD. Values from two control lines as well as two *SNCA^Dupl^* lines and three independent differentiation rounds per cell line were used for the quantification (*n* = 3/line). Statistics: Mann–Whitney test; * *p* < 0.05.

In our previous study, we demonstrated a significant reduction in bTubIII in hiPSC-derived mDANs from another *SNCA^Dupl^* patient [13], therefore we also analyzed bTubIII levels in NPCs and mDANs derived from *SNCA^Dupl^* hiPSC lines enrolled in this study by WB. By using the same neuronal differentiation protocol, we again detected a significant decrease in bTubIII levels in *SNCA^Dupl^* mDANs differentiated for 10 days compared to control mDANs (Figure 4A,B, average 0.8 fold decrease, * *p* < 0.05, Supplementary Figure S4A). This effect was remarkably stronger in mDANs at a more advanced differentiation stage (differentiated for 24 days, Supplementary Figure S4B, average 0.6-fold decrease, * *p* < 0.05). Notably, bTubIII downregulation was only observed in mDANs. No significant differences in bTubIII levels were present between control and *SNCA^Dupl^* NPCs (Figure 4A,B). Analysis of bTubIII mRNA levels via reverse transcription polymerase chain reaction (RT-PCR) did not significantly differ between control and *SNCA^Dupl^* cells at any differentiation stage (Supplementary Figure S4C), suggesting that the loss of bTubIII occurs at the protein level. In addition to bTubIII, we also detected a significant reduction in the acetylated aTub level in mDANs (Figure 4C,D). Furthermore, a significant increase in beta actin (bActin), a further essential molecular component of the cytoskeleton, was detected in *SNCA^Dupl^* mDANs (Figure 4E,F). In summary, diminished levels of microtubule building elements and elevated levels of bActin suggest a specific dynamic rearrangement pattern of the cytoskeleton during PD-related neuronal differentiation. 

### 2.5. bTubIII Is Redistributed in SNCA^Dupl^ mDANs

**Figure 5:**
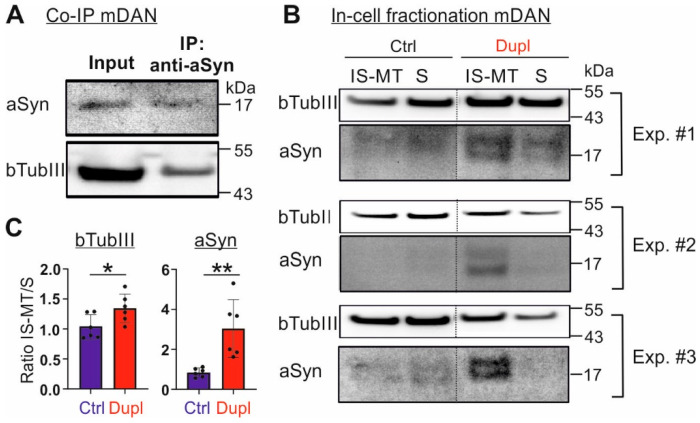
Interaction of aSyn and bTubIII and their reorganization in *SNCA^Dupl^* mDANs. (**A**) WB analysis of immunoprecipitation of aSyn from hiPSC-derived mDANs differentiated for 10 days by using an anti-aSyn antibody (Syn1). bTubIII is co-precipitated with aSyn. (**B**) In-cell fractionation of control and *SNCA^Dupl^* mDANs and WB analysis of aSyn and bTubIII in soluble (S) and insoluble, microtubule-associated (IS-MT) fractions. Representative WB images of three experiments (#1, #2, #3) with mDANs from one control and one *SNCA^Dupl^* hiPSC cell line are shown. Note, due to a strong dilution effect after immunoprecipitation and in-cell fractionation, transferred aSyn on the blots shown in (**A**,**B**) was only visible by loading the maximum volume onto SDS-PAGE gels and using the SuperSignal™ West Femto Maximum Sensitivity Substrate kit (Thermo Fisher Scientific). (**C**) For quantification, the ratio of bTubIII or aSyn in the IS-MT versus the S fraction was calculated. Values from one control line as well as one *SNCA^Dupl^* line and three independent differentiation rounds per cell line were used for the quantification (*n* = 3/line). aSyn overexpression in hiPSC-neurons leads to a shift of aSyn and bTubIII into the IS-MT pool. Statistics: unpaired t-test; * *p* < 0.05; ** *p* < 0.01.

In mDANs, we also performed immunoprecipitation of aSyn and were able to detect bTubIII in immunoprecipitated produces, thereby confirming the interaction of aSyn with bTubIII in the hiPSC-derived neuronal cell system (Figure 5A). After having confirmed elevated aSyn levels and aSyn-bTubIII interaction in *SNCA^Dupl^* patient-derived cells, we next asked whether the effect of aSyn overload on redistribution of aSyn and microtubule elements observed in the H4-aSyn cell model (Figure 2) can be recapitulated in patient-derived mDANs. Applying in-cell fractionation as depicted in Figure 2A, we again observed elevated aSyn levels in insoluble, microtubule-associated fractions from *SNCA^Dupl^* mDANs compared to the levels of control mDANs (Figure 5B,C), indicating an increased aggregation of aSyn. To further corroborate the association of aSyn overload with its aggregation, we analyzed aSyn aggregation in NPCs by a solubility assay, in which soluble and insoluble aSyn were separated by centrifugation at 100,000× *g* for 1 h. We observed an increase in insoluble aSyn in *SNCA^dupl^* NPCs compared to control NPCs (Supplementary Figure S5), suggesting that aSyn aggregation already occurs at the NPC stage. 

Most importantly, in-cell fractionation revealed a proportional increase in the microtubule element, bTubIII, in the insoluble fraction extracted from *SNCA^Dupl^* mDANs compared to control mDANs (Figure 5B,C). Again, these data support a reorganization of the microtubule elements in patient-derived neuronal cells with an abnormal increase in aSyn levels.

### 2.6. Increased aSyn Perturbs Neurite Morphology Already in an Early Differentiation Stage 

**Figure 6:**
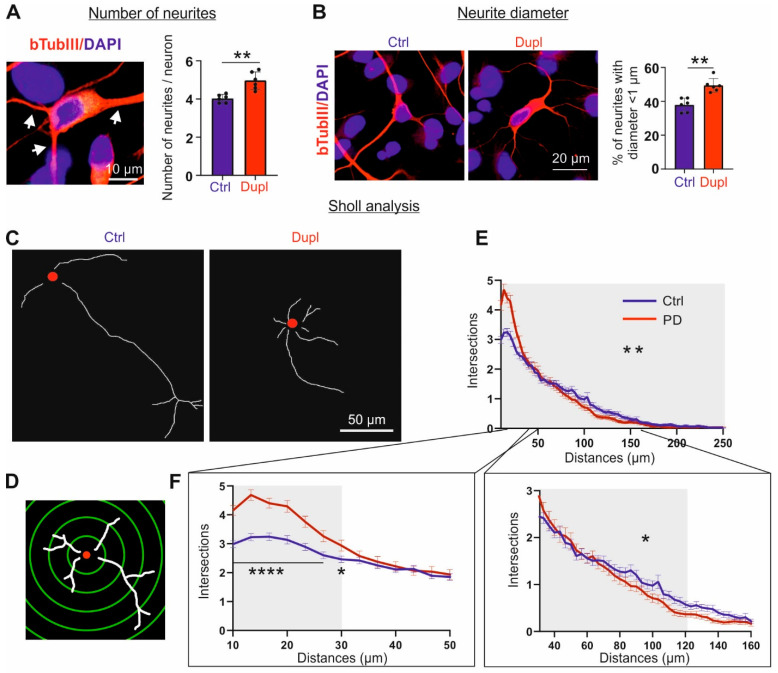
Analysis of neurite morphology of neurons differentiated from hiPSCs. (**A**) An example neuron (**left**) for the analysis of primary neurite numbers per cell (**right**). Neurite numbers of bTubIII positive neurons were counted using the “Cell counter” plugin within ImageJ software. The *SNCA^Dupl^* neurons show more primary neurites per cell than the control neurons. Total *n* > 800 neurons in twelve images per condition were examined. (**B**) Representative images of bTubIII+ neurons (red) derived from the control and *SNCA^Dupl^* hiPSC. Neurite diameter was examined by measuring the diameter of each neurite, where it leaves the cell body using “Straight Line” tool within ImageJ. Quantification shows an increase in thin neurites with a diameter <1 µm in *SNCA^Dupl^* neurons compared to control neurons. Microscope images with at least 800 neurites per condition were examined. For the quantification shown in A and B, average values from two control lines as well as two *SNCA^Dupl^* lines and three independent differentiation rounds per line were used and are shown in mean ± SD (*n* = 3/line). Statistics: Mann–Whitney test; ** *p* < 0.01. (**C–F**) Sholl analysis of control and *SNCA^Dupl^* neurons. (**C**) Representative micrographs of skeletonized neurons from a control and *SNCA^Dupl^* neuron, respectively. The nuclei are highlighted by red solid circles and resemble the center for Sholl analysis on a single cell level. (**D**) Scheme of Sholl analysis of a skeletonized neuron with neurites (white) and superimposed centric circles (green). The radius interval between circles was 3.3 µm per step, ranging from 10 to 250 µm from the center of the neuronal nuclei (red). (**E**) The numbers of neurite intersections show a significantly stronger decrease in *SNCA^Dupl^* neurites compared to the control neurons. For quantification, the number of intersections per neuron was counted. Mean ± SEM from *n* = 120 control and *SNCA^Dupl^* neurons, respectively, are shown. For statistics, the decrease in intersections per cell was determined by the slope decrease in intersections per cell ranging from 10–250 µm (grey) and the average slope difference between control and PD neurons (120 neurons, respectively) were evaluated using unpaired t-test (** *p* < 0.01). (**F**) Left: In the near proximity of the soma (distance range 10 to 30 µm, grey), the number of neurite intersections in *SNCA^Dupl^* neurons is significantly higher than in the control neurons, indicating an increase in short neurites in *SNCA^Dupl^* neurons. Statistics: two-way ANOVA; * *p* < 0.05, **** *p* < 0.0001. Right: at the long distance range from the soma (30 to 120 µm, grey), the number of neurite intersections in *SNCA^Dupl^* neurons is lower as compared to control neurons, indicating a decrease in long neurites in *SNCA^Dupl^* neurons. For statistics, the decrease in intersections per cell was determined by the slope decrease in intersections per cell ranging from 30 to 120 µm and the average slope difference between control and PD neurons (120 neurons) was evaluated by unpaired t-test (* *p* < 0.05).

In primary rat midbrain neurons, forced aSyn overexpression via viral transduction has been shown to affect neurite morphology and axonal transport activity [8]. Here, we questioned whether patient-derived *SNCA^Dupl^* mDANs, with a clinically relevant aSyn overload, also display a neuritic phenotype. We focused on mDANs differentiated for 10 days, an early differentiation stage, as significantly greater differences in aSyn (Figure 3) and tubulin (Figure 4) levels in control and *SNCA^Dupl^* mDANs, as well as a redistribution of aSyn and bTubIII in *SNCA^Dupl^* mDANs (Figure 5) were already observed at this stage. Analysis of the number of primary neurites, projections emanating directly from the cell body, showed a significant increase in primary neurites in *SNCA^Dupl^* carrying neurons (Figure 6A). Thickness measurements revealed an increase in neurites with a diameter < 1 µm in neurons carrying the *SNCA^Dupl^*, indicating a reduction in neurite thickness in such neurons (Figure 6B). Furthermore, the analysis of neurite outgrowth and length via Sholl analysis (Figure 6C,D) showed a significant increase in the number of neurite intersections in the near proximity of the soma (distance to the soma ranging from 10–30 µm) (Figure 6C,E,F left), supporting the conjecture that *SNCA^Dupl^* neurons exhibit a greater number of short neurites when compared with control neurons. By contrast, the number of neurite intersections within the long-distance range (30 to 120 µm from the soma) decreased in *SNCA^Dupl^* neurons (Figure 6C,E,F right). In summary, neurites developed from *SNCA^Dupl^* mDANs exhibit an increased neurite initiation. However, neurite outgrowth appears to be hampered in *SNCA^Dupl^* neurons, characterized by diminished length and thickness in contrast to those of control neurons.

## 3. Discussion

In this study, we investigate the impact of aSyn on microtubule organization and neuritic integrity using aSyn overexpressing H4 cells and hiPSC-derived neuronal cells generated from a PD patient carrying a heterozygous duplication of the *SNCA* locus. Our data suggest a mechanistic link between aSyn overexpression, aSyn aggregation, altered aSyn-tubulin interaction and microtubule organization, as well as impaired neuritic integrity in patient-derived neurons.

### 3.1. aSyn Overload and Its Aggregation

Although the aetiology of PD may be multifactorial, elevated aSyn levels are assumed to be an important risk factor for aSyn aggregation. This is particularly supported by genetic evidence that *SNCA* duplication or triplication alone is sufficient to cause PD [20,21]. Extending our previous studies on hiPSC-derived neurons (CPNs and mDANs from *SNCA^Dupl^* PD patients [9,13], we confirm increased aSyn protein levels in hiPSC-derived cells carrying *SNCA^Dupl^*. An approximately two-fold increase in aSyn protein levels was clearly detected in NPCs or mDANs differentiated for 10 days (Figure 3), thereby verifying our previous findings in hiPSC-derived neurons generated from other *SNCA^Dupl^* patients [9,13]. Importantly, we demonstrate a clear link between a high aSyn level and aSyn aggregation in two aSyn overexpressing H4 cell lines (Figure 1A), *SNCA^Dupl^* NPCs (Figure 3 and Supplementary Figure S5) and mDANs (Figure 3 and Figure 5B). aSyn overload-mediated accumulation of aSyn aggregated species can be explained by overwhelmed protein degradation pathways and/or increased resistance to protein degradation machineries, as indicated by numerous studies [22,23,24,25]. 

### 3.2. aSyn and Microtubule Organization

The interference of aSyn with the microtubule network has been implied by several in vitro and in vivo studies, demonstrating that aSyn interacts physiologically directly with tubulin in Hela cells and in hamsters’ brain homogenates via co-immunoprecipitation assays (reviewed in [15]). In the present study, we observed a high binding affinity between aSyn and bTubIII in aSyn overexpressing H4 cells and in *SNCA^Dupl^* mDANs. More importantly, unbiased proteomic analysis revealed that aggregated aSyn, induced by aSyn overexpression, exhibits a higher binding capacity for a number of beta tubulin isoforms, compared to non-aggregated aSyn. These findings highlight the role of aSyn aggregation in the dysregulation of tubulin homeostasis. Our findings in aSyn overexpressing H4 cells and *SNCA^Dupl^* mDANs suggest that tubulin dyshomeostasis occurs probably at two different levels in neurons: 

Firstly, aSyn impacts tubulin levels, including bTubIII and acetylated aTub (Figure 2D,F and Figure 4), either directly or indirectly. A downregulation of bTubIII was observed in the present study and in our previous study, in which mDANs differentiated from an additional *SNCA^Dupl^* PD patient were characterized [13]. Interestingly, NPCs and H4-aSyn cells did not show a decrease in bTubIII, despite increased aSyn (Figure 1A and Figure 3) and aggregation (Figure 1A and Figure 2B, and Supplementary Figure S5) as well as a reorganization of microtubule elements (Figure 2). Thus, our current data suggest a neuronal differentiation-dependent decrease in bTubIII protein levels, which are linked to *SNCA^Dupl^* mDANs. Interestingly, the downregulation of tubulin elements was not described in other aSyn overexpression cell models, for example, in hiPSC-neurons with *SNCA* triplication [21,26]. It should be noted that tubulins are involved in a dynamic balance between microtubule assembly and disassembly processes and that their solubility is closely correlated to assembly extent, as indicated in Figure 2 and Figure 5. Thus, the fact that tubulin downregulation was not observed in other cell models might be attributed to the heterogeneity of cell models, differentiation protocols or biochemical processing of samples (e.g., centrifugation conditions for sample preparation). The mechanism by which aSyn regulates tubulin levels remains unclear. The lack of alteration in bTubIII transcripts, as determined in this study, suggests a posttranscriptional regulatory mechanism.

Secondly, aSyn interferes with microtubule organization. The regulatory role of aSyn in microtubule formation has been implied in several, predominantly cell-free in vitro studies (reviewed in [15,27]). However, the question as to how aSyn modulates tubulin polymerization remains controversial. While some studies reported that aSyn promotes tubulin polymerization in vitro, other studies provided data for an inhibitory effect. Our previous in vitro study indicated that the aggregation states of aSyn and microtubule-associated proteins have a great impact on microtubule assembly: For example, wild type aSyn aggregates reduced microtubule assembly in the presence of Tau [11]. A detailed cell-free study by Cartelli et al. recently suggested a dual role of aSyn in regulating microtubule assembly [12]: Physiologically, aSyn binds to α2β2 tubulin tetramers and thereby acquires alpha helical structures, thus promoting furthermore microtubule nucleation. Pathologically, PD-linked, aggregation-prone aSyn mutants are not able to undergo tubulin–mediated conformational changes and thereby cause tubulin aggregation rather than organized polymerization. Hence, the correct organization of the microtubules could be impaired by aggregated aSyn. Interestingly, our results from the experiments on human cell model systems (Figure 2 and Figure 5) support these cell-free findings. In both hiPSC *SNCA^Dupl^* mDANs and H4-Syn cells, we observed increased insoluble aSyn levels, correlating with aSyn overexpression, accompanied by a shift of soluble bTubIII into insoluble fractions. Although it is currently difficult to distinguish abnormally aggregated tubulins from the assembled tubulins, our data regarding impaired neurite outgrowth in *SNCA^Dupl^* mDANs support the notion that increased aSyn levels and aggregation result in an improper aggregation of tubulins. Future in-depth studies employing more advanced biochemical and ultrastructural imaging techniques would be necessary to address the issue of aSyn conformer-dependent tubulin alterations under pathological conditions. 

### 3.3. aSyn and Neuritic Integrity

Loss of dopaminergic neurons in the SNpc is the main cellular determinant for movement disorders as observed in PD patients. By the time first motor symptoms occur, 30–70% dopaminergic neurons in the SNpc are already degenerated [28,29]. Therefore, understanding the mechanisms underlying early neurodegenerative processes is a focus point in PD research for delaying onset of motor symptoms and halting disease progression.

Neuritic degeneration in affected neurons has been suggested to be a common initial pathological event in PD and other neurodegenerative disorders [6,30]. Specifically in PD, post-mortem studies of PD brains demonstrate a significant decrease in dopaminergic fibers, for example, in the dorsal putamen, while dopaminergic cell bodies in the SN were less affected [31,32]. These findings were corroborated by in vivo animal studies using imaging tools, demonstrating an early proportional loss of striatal and putaminal dopaminergic terminals (reviewed in [30,33]). 

In order to understand the effect of aSyn on neuritic deficits, Koch et al. [8] and Oliveira et al. [26] investigated neurite morphology in primary midbrain neuron overexpressing aSyn induced via viral transduction and in hiPSC neurons carrying *SNCA* triplication, respectively. These studies showed that aSyn overexpression remarkably reduces the elongation of processes [8,26] and impairs branching behavior [8]. Here, we provide consistent results that *SNCA^Dupl^*-mediated aSyn elevation hampers the neuritic development (Figure 6). This was reflected by reduced neurite length and diameter. Strikingly, the number of neurites directly originating from the soma was significantly greater in *SNCA^Dupl^* neurons, applying two different assessment approaches, i.e., counting the primary neurites and via the Sholl analysis. An identical morphological change was described in the study by Koch et al. on rat primary neurons [8]. They speculated that increased primary neurites may be a result of dysregulated cytoskeletal homeostasis. Whereas neurite initiation is modulated by actin assembly, neurite elongation is dependent on tubulin polymerization [8]. Supporting that hypothesis, we observed that elevated aSyn in *SNCA^Dupl^* neuronal cells correlates with decreased bTubIII and acetylated aTub, but increased bActin levels. Indeed, the effect of aSyn on actin structure and assembly dynamics has been previously shown in cell-free conditions and in cell models [34]. 

Recent accumulating evidence indicates a pivotal role of prion-like intercellular propagation of aSyn in the pathological progression of PD. Numerous studies, either on patient post-mortem tissue [35,36,37] or on animal models [38,39], have provided supportive evidence for aSyn propagation pathways from the periphery to the central nervous system (CNS), e.g., from the gut to the CNS via the vagus nerve, followed by a stereotypic propagation within the CNS, i.e., from the olfactory bulb and the brain stem to the striatum and cortex through the substantia nigra. Neuron-to-neuron, trans-synaptic spreading of aggregated aSyn has been suggested as an underlying mechanism of aSyn propagation [40]. Functional axonal transport of aSyn within the neurons seems to be required for such trans-synaptic spreading [41]. Interestingly, our present data and previous results strongly support impairments in neuritic integrity and a dysfunctional axonal transport in diseased neurons [9]. One explanation, as speculated by Lamberts et al. [42], could be that the recipient neuron takes up smaller aSyn aggregates, and those could be transported to the synaptic terminal with still functional axonal transport machinery. During this process, lager aggregates could form by recruiting endogenous aSyn. Based on our findings, we further speculate a role of distinct spatial interaction of aSyn and the microtubule network in trans-synaptic spreading of aSyn pathology. In addition to the intercellular transfer property, recent studies have also demonstrated the existence of distinct aSyn strains, which are distinguished by differences in biochemical properties as well as transmission potency, and furthermore are related to specific phenotypes and different synucleinopathies, such as PD and multiple system atrophy [43,44,45,46,47]. Our previous cell-free study has shown that aSyn oligomers, fibrils and monomers divergently impact microtubule assembly and the interaction of microtubules with anterograde motor protein kinesin [11]. Therefore, it is conceivable that distinct aSyn strains may interfere with the microtubule network differently. Further studies are required to address strain and axonal transport-associated interaction of aSyn and microtubule machinery. 

In conclusion, we demonstrate in human cell models, in particular in *SNCA^Dupl^* patient-derived neuronal cells with a clinically relevant aSyn level, that aSyn overload induces an elevation in aggregated aSyn. We provide the evidence that aSyn binds directly to bTubIII in both aSyn overexpressing H4 cells and hiPSC-derived neuronal cells. More importantly, aggregated aSyn preferentially interacts with tubulins. Furthermore, abnormal aSyn overexpression not only promotes its own aggregation, but also increases insoluble tubulin levels, correlating with altered neuritic phenotypes in patient-derived neurons. Given the important role of neuritic degeneration in neuronal death, our data support the mechanistic pathway from aSyn overload to neuritic impairment via the disruption of microtubule organization. Thus, pharmacological interference, with regard to PD-linked neuritic degeneration, might target the maintenance of microtubule organization/homeostasis in future. 

Models utilizing hiPSC-derived cells offer unique advantages, because of restrictions regarding the use and availability of human brain tissue and the limitations of working with animal models due to differences between humans and animals. In particular, hiPSC neurons differentiated from patients with monogenic forms of PD, as presented in this study and other published studies (reviewed in [48,49]), recapitulate important, although not fully, PD-linked phenotypes. Despite of the lack of some epigenetic or microenvironmental effects, e.g., caused by aging or neuron-glia network, genetic effect may have a dominant role in hiPSC-derived neurons carrying PD-causing mutations. Therefore, such models present as a promising candidate for application in PD diagnosis, monitoring disease progression and evaluation of novel therapeutic options due to the minimally invasive nature of cell harvesting. 

## 4. Materials and Methods

### 4.1. H4 Cell Lines

Naïve H4 human neuroglioma cells (ATCC, HTB-148), a H4 cell line overexpressing wild type aSyn (H4-aSyn, generated as described in Menges et al. [50]) and a H4 cell line overexpressing wild type aSyn under the control of a tetracycline inducible promoter (H4-aSyn tet-off, as previously described in Mazzulli et al. [51]), were used in this study. The cells were either cultured in Opti-MEM™ Reduced Serum Medium, GlutaMAX™ Supplement (Thermo Fisher Scientific, Waltham, MA, USA) supplemented with 10% FCS (Sigma Aldrich, Munich, Germany) and 1% pen-strep (Thermo Fisher Scientific) for naïve and H4-aSyn cells or in OptiMEM media (Thermo Fisher Scientific) containing 5% FCS, 200 µg/mL geneticin (Thermo Fisher Scientific), 200 µg/mL hygromycin (Sigma Aldrich), and 1% pen-strep for H4-aSyn tet-off cells. For the experiment, cells were seeded at 2 × 10^5^ cells per well in a 6-well plate or 1 × 10^6^ cells per 10 cm^2^ dish and grown until the cells were confluent. The expression of aSyn in H4-aSyn tet-off cells was turned off by the addition of 2 µg/mL doxycycline (Sigma Aldrich) (H4-aSyn tet-off+Dox) for 24–72 h. 

### 4.2. Human iPSC

hiPSCs derived from human dermal fibroblasts were obtained from the stem cell core unit of the Friedrich-Alexander Universität Erlangen-Nürnberg (FAU). Written informed consents were received from voluntary donors of skin biopsies prior to inclusion in the study at the Movement Disorder Clinic at the Department of Molecular Neurology, Universitätsklinikum Erlangen (Erlangen, Germany). 

All experiments using hiPSC-derived cells were conducted in accordance with the Institutional Review Board Approval (Nr. 259_17B). Skin biopsy samples were obtained from a PD patient carrying *SNCA^Dupl^* and two healthy individuals with no history of neurologic disease. The PD patient is female, was 44 years old at the time of skin sample collection, and presented with the symptoms of disease manifestation at age 39. The occurrence of the *SNCA^Dupl^* was confirmed using next generation sequencing, revealing a heterozygous duplication of *SNCA*. A 69-year-old male donor (#1) and a 42-year-old female donor (#2) were recruited as control persons at the time of taking biopsy. hiPSCs were reprogrammed from fibroblasts as described previously [9,13,52]. Two PD-hiPSC lines (UKERi7GG-S1-004 and –S1-005) derived from the PD *SNCA^Dupl^* patient and two control hiPSC lines (UKERiG3G-R1-039 from control #1 and UKERi1JF-R1-011 from control #2, respectively) were included in this study. 

### 4.3. Differentiation of mDANs

mDANs were generated utilizing a small molecule-induced hiPSC differentiation protocol according to Reinhardt et al. [17] and our previous studies [13,19], adapted with minor modifications (Supplementary Figure S3A): for the induction of NPCs, hiPSCs were resuspended in mTeSR+ Medium (STEMCELL Technologies, Vancouver, BC, Canada) supplemented with the small molecules 1 μM LDN, 10 μM SB-431542, 3 μM Chir, 0.5 μM Purmorphoamine (PurMA) as well as 10 µM ROCK inhibitor, and cultured on an Ultra-Low Adhesion Surface Plate (Corning, New York, NY, USA). On the next day, the medium was replaced with the same medium supplemented with small molecules as prior; however, the use of ROCK inhibitor was excluded. After another day, the medium was replaced with N2B27 medium (50% DMEM/F12, 50% Neurobasal Medium, 1:200 N2 Supplement, 1:100 B27 Minus Vitamin A, 1:200 GlutaMAX^TM^ Supplement and 1:100 pen-strep (all from Thermo Fisher Scientific) supplemented with the aforementioned small molecules) for two additional days (total 4 days). The medium was thereafter replaced with smNPC medium (N2B27 medium supplemented with 3 μM Chir, 0.5 μM PurMA and 150 µM ascorbic acid). After two more days, the resulting embryoid bodies (EBs) were triturated and replaced on a Matrigel (Corning)-coated plate in smNPC medium supplemented with ROCK inhibitor, which was removed on the following day. Afterwards, a medium replacement was conducted every other day using smNPC medium. NPCs were passaged every six days at a ratio of 1:5 using Accutase (Sigmal-Aldrich). After a minimum of five passages, the NPCs were differentiated into mDANs. For this, NPCs cultured in smNPC medium for two days were further cultured in N2B27 medium supplemented with 100 ng/mL FGF8, 1 μM PurMA and 200 μM ascorbic acid. After eight days, the cells were replated on a Matrigel-coated plate from day 9 with maturation medium (N2B27 supplemented with 10 ng/mL BDNF, 10 ng/mL GDNF, 1 ng/mL TGFb3, 200 μM ascorbic acid, 500 μM Dibutyryl-cAMP (dbcAMP) and ROCK inhibitor). The cell density for plating was either 1.5 × 10^6^ per well for a 6-well plate or 1.5 × 10^5^ per well for a 24-well plate. After one day, the medium was replaced with maturation medium without ROCK inhibitor and the cells were incubated for another day. Finally, the medium was replaced with maturation medium without PurMA. mDANs were cultured in this maturation medium with medium replacement twice per week.

### 4.4. Dot Blot 

Native dot blot analysis of aggregated aSyn was performed by applying the cell lysate containing 10 µg total protein on a nitrocellulose membrane (0.45 µm, Buckinghamshire, UK) in a total volume of 5 µL. Membrane was air-dried for 3 h and subsequently blocked in 5% non-fat dry milk in TBS for 1 h at RT. Aggregated aSyn was probed by using a rabbit conformation-specific antibody MJFR-14-6-4-2 (Abcam, Cambridge, UK) in combination with IRDye 800CW donkey anti-rabbit secondary antibody (LI-COR Biosciences, Lincoln, NE, USA) using the identical immunostaining protocol for WB. Fluorescent signals were detected by using the Odyssey imaging system (LI-COR Biosciences). Loading of total protein was controlled by staining total protein loaded using direct blue 71 according to Hong et al. [53]. For this purpose, the blot membrane was stained by using a working solution of direct blue 71 containing 0.008% direct blue 71 (Sigma Aldrich), 40% Ethanol and 10% acetic acid for 5 min at RT, followed by rinsing the membrane with 40% ethanol and 10% acetic acid. 

### 4.5. Western Blot (WB)

Cells were homogenized in TBS containing 1% Triton X 100 using a B. Braun Potter S Homogenizer (Sartorius AG, Göttingen, Germany). Homogenates were thereafter diluted in RIPA buffer (50 mM Tris/HCl pH 8.0, 150 mM NaCl, 5 mM EDTA, 1% NP40, 0.5% sodium deoxycholate, 0.1% SDS) for 30 min on ice to lyse the cells. Subsequently, samples were centrifuged at 10,000× *g* for 10 min at 4 °C. Protein content of the supernatant was determined using a bicinchoninic acid (BCA) assay (Thermo Fisher Scientific). For SDS-PAGE, 15 μg total protein was mixed with LDS-sample buffer and reducing agent (Thermo Fisher Scientific) according to the manufacturer’s recommendations, and boiled afterwards at 70 °C for 10 min. For electrophoresis, samples were loaded on precast gels NuPage 4–12% Bis-Tris or Bolt 4–12% Bis-Tris Plus (Thermo Fisher Scientific). For protein transfer, the gel was blotted on a PVDF membrane (Merk Millipore, Darmstadt, Germany). After blotting, the PVDF membrane was incubated with 4% PFA for 15 min, then thoroughly rinsed with TBS-T. For protein loading control, the membrane was stained using Ponceau S solution (Sigma Aldrich) prior to the immunodetection. 

For immunostaining, the membrane was incubated with primary antibodies either for 1 h at RT or overnight at 4 °C, followed by incubation of horseradish peroxidase-conjugated secondary antibodies. Specific proteins were visualized with SuperSignal West Pico Plus or SuperSignal West Femto (both from Thermo Fisher Scientific) chemiluminescent horseradish peroxidase substrate. Signal detection and imaging were performed by using Fusion Fx7 (PEQLAB). Signal intensities were measured by using Image Lab Software (Version 6.0.1, BioRad) or Bio1D (PEQLAB). Only images not oversaturated were used for WB analysis and quantification. 

### 4.6. Immunoprecipitation and LC-MS Analysis

To verify the interaction of aSyn and specific microtubule proteins, immunoprecipitation was conducted by using the mouse anti-aSyn antibody Syn1. Cells were homogenized in TBS buffer or TBS buffer with 1% Triton X 100, 1% NP40, or RIPA (all supplemented with protease-inhibitor cocktail (cOmplete tablets)) using a Potter S homogenisator (B. Braun, Melsungen, Germany) in the concentration recommended by the manufacturer. The cell homogenates were centrifuged at 500× *g* at 20 °C for 5 min to remove cell debris. The protein concentration of the supernatant was determined via BCA assay and adjusted to 1 µg/µL using the buffer for cell lysis. Prior to co-immunoprecipitation, 10 µL Protein G Plus/Protein A agarose beads (IP05, Calbiochem, San Diego, CA, USA) were incubated with 5 µL Syn1 antibody at 4 °C for 2 h under rotation, followed by the addition of 50 µL cell lysate to the antibody and agarose bead mixture. The immune complex was incubated overnight at 4 °C under continuous rotation and centrifuged at 2000× *g* at 4 °C for 10 min. The pelleted beads were rinsed three times with 100 µL cold lysis buffer. Bound proteins were eluted by using 1× LDS-sample buffer (Thermo Fisher Scientific), subjected to SDS-PAGE and analyzed by immunoblotting. 

For identification of proteins bound to aSyn via LC-MS, a mouse anti-aSyn antibody Syn211 (Sigma Aldrich) and the conformation-specific MJFR-14-6-4-2 antibody were used. Cells were lysed in 40 mM HEPES buffer pH 7.4 containing 120 mM NaCl, 1 mM EDTA, 0.3% CHAPS and 10% glycerol. After removing cell debris by centrifugation at 21,000× *g* for 10 min at 4 °C, the protein concentration was determined via a BCA protein assay. Immunoprecipitation was performed using cell lysate with 1000 µg total protein, incubated with protein A/G beads (Santa Cruz, Dallas, TX, USA) for 30 min at 4 °C under agitation. After removal of the beads via centrifugation at 1000× *g* for 5 min at 4 °C, the supernatant was either incubated with 3 µL Syn211 or MJFR-14-6-4-2 overnight at 4 °C under rotation. Simultaneously, 30 µL protein A/G beads were blocked in 2% freshly prepared BSA overnight at 4 °C under rotation and washed by 500 µL lysis buffer three times by centrifugation at 1000× *g* for 5 min. Next, the blocked protein A/G beads were added to the immune complex for 4 h at 4 °C under rotation. The immunoprecipites were collected via centrifugation at 1000× *g* for 5 min at 4 °C and washed three times with the lysis buffer. Bound proteins were eluted by 1× sample loading dye containing 22 mM Tris/HCl (pH 6.8), 9% (*v*/*v*) glycerol, 0.02% SDS (*v*/*v*), 22.4 mM DTT and bromophenol blue for mass spectrometric identification of proteins co-precipitated via Syn211 or MJFR-14-6-4-2 antibody. 

Following the elution from protein A/G beads, samples were made up to 100 µL with ammonium bicarbonate (ABC, 100 mM) in 1% SDS and reduced with tris(2-carboxyethyl)phosphine (5 mM) at 65 °C for 30 min and then alkylated in the dark at 25 °C using iodoacetamide (12.5 mM). Proteins were precipitated on Speedbead Magnetic Carboxylate modified beads (SP3 beads, 10:1, beads to protein, GE healthcare, Chicago, IL, USA) by adding a 6-fold volume of ethanol. The samples were incubated with the beads for 20 min to initiate binding and then washed three times with 80% ethanol. Samples were resuspended in ABC (100 mM) and trypsin (at an enzyme:protein ratio of 1:25) added and left to digest overnight at 37 °C. The supernatant was removed using a magnet and then dried via vacuum centrifugation and stored at −20 °C before being resuspended in running buffer (3% acetonitrile (ACN), 0.1% trifluoroacetic acid (TFA)) prior to LC-MS measurements.

LC-MS analysis was conducted using a Dionex Ultimate 3000 nano-UHPLC coupled with an Orbitrap Fusion Lumos mass spectrometer (Thermo Fisher Scientific, Bremen, Germany). The samples were washed on a trap column (Acclaim Pepmap 100 C18, 5 mm × 300 μm, 5 μm, 100 Å, Dionex) for 4 min with 3% ACN/0.1% TFA at a flow rate of 30 μL/min prior to peptide separation using an Acclaim PepMap 100 C18 analytical column (50 cm × 75 μm, 2 μm, 100 Å, Dionex). A flow rate of 300 nL/min using eluent A (0.05% formic acid (FA)) and eluent B (80% ACN/0.04% FA) was used for gradient separation. Spray voltage applied on a metal-coated PicoTip emitter (10 μm tip size, New Objective, Woburn, MA, USA) was 1.8 kV, with a source temperature of 275 °C. Full scan mass spectrometry (MS) spectra were acquired between 350 and 1400 *m*/*z* at a resolution of 120,000 at *m*/*z* 400. The 10 most intense precursors with charge states greater than 2+ were selected with an isolation window of 1.4 *m*/*z* and fragmented by HCD with normalized collision energies of 30 at a resolution of 30,000. Lock mass (445.120025) and dynamic exclusion (30 s) were enabled. Samples were measured in duplicate (technical injection replicates).

For MS data analysis, the MS raw files were processed by Proteome Discover 2.2 (Thermo, version 2.2.0.388) and MS/MS spectra were searched using the Sequest HT algorithm against a database containing common contaminants (45 sequences) and the canonical human database. The enzyme specificity was set to tryptic with two missed cleavages allowed. A MS1 tolerance of 10 ppm and a MS2 tolerance of 0.02 Da were implemented. Oxidation (15.995 Da) of methionine residues was set as a variable modification, while carbamidomethyl (57.02146 Da) on cysteine residues was set as a static modification. Minimal peptide length was set to 6 amino acids and the peptide false discovery rate (FDR) was set to 1%. Label free quantitation was performed in Proteome Discover using Minora Feature Detector. The files were then exported and a statistical analysis was performed using Perseus (1.6.10.43). Samples were log2 transformed and filtered so that only proteins that were observed in both biological samples were kept. A two-tailed t-test was performed using a permutation based false discovery rate (FDR) to account for the multiple testing hypothesis (technical injection replicates were preserved during randomization). Candidates were filtered using an FDR of 1% and a fold change (s0) of 2.

### 4.7. In-Cell Fractionation

Fractionation of cells to soluble (S) and detergent resistant, insoluble, microtubule-associated fractions (IS-MT) was performed according to Katsetos et al. [16] with minor modifications (Figure 2A). Briefly, attached H4 cells (naïve or H4-aSyn cells) or hiPSC-mDANs were rinsed with prewarmed (37 °C) microtubule stabilizing buffer MSB I (100 mM MES/KOH, pH 6.9, 2 mM EGTA, 2 mM MgCl_2_). The cells were incubated in pre-warmed MSB I containing protease-inhibitor (cOmplete tablets, Roche, Basel, Swiss) and 0.2% Triton X 100 (MSB II) for 1 min at 37 °C. Afterwards the soluble fraction of cells was carefully collected from the cell culture well and centrifuged at 5000× *g* for 1 min at RT. The soluble fraction (S) and pellet (P) obtained were separated and kept. The insoluble structure remaining in the well was solubilized with MSB II + LDS-sample buffer and pooled with the pellet (P), generating insoluble, microtubule-associated fraction (IS-MT). Finally, both S and IS-MT fractions were adjusted to the equal volume using MSB II buffer and mixed with LDS-sample buffer for SDS-PAGE and WB.

### 4.8. Immunocytochemistry (ICC)

Cells were treated with 2% paraformaldehyde (PFA) for 8 min, followed by another 10 min fixation in 4% PFA and three rinses with PBS. Thereafter, cells were blocked for 15 min with fish skin gelatin buffer (Tris-buffered saline (TBS) with 0.4% cold water fish skin gelatin in water, 1% bovine serum albumin (BSA) and 0.1% Triton X 100). Subsequently, cells were incubated with a primary antibody overnight at 4 °C followed by incubation with a secondary, fluorescence-labelled antibody for 1 h at room temperature (RT). Finally, cell nuclei were stained with DAPI for 15 min at RT. Images were captured using an Axio Observer inverted fluorescence microscopes (Carl Zeiss AG, Oberkochen, Germany).

### 4.9. Analysis of Neuronal and Neurite Morphology

Neuronal and neuritic morphology was analyzed using the “Sholl Analysis” plugin within the software ImageJ (FIJI distribution, v1.53c, (37)). mDANs after 10 days of differentiation were fixed and immunostained using an anti-bTubIII antibody as described in the ICC section. Images were further processed using the “Simple Neurite Tracer (SNT)” of “NeuroAnatomy” Plugin in ImageJ. Five bTubIII+ neurons were traced on each of 12 images per cell clone, from three differentiation experiments, respectively. Skeletonized neurons processed using SNT, with marked neurites of one neuron and the localization of its nucleus, were saved in individual files. The nucleus served as the center for Sholl analysis. Sholl analysis was performed using the “Sholl” plugin (v4.0.1) in ImageJ. Shell parameters were set to 10 µm start radius, 3.33 µm step size and 250 µm end radius. The number of primary neurites was counted using “Cell counter” plugin in ImageJ. Neurite diameter was examined using the “Straight Line” tool in ImageJ to measure the diameter of primary neurites, using the cell body as a reference point. Measurements were subsequently grouped in diameters ≥1 µm and <1 µm. 

### 4.10. Antibodies

All antibodies utilized for WB and ICC and their dilutions are summarized in Table 1.

### 4.11. Statistical Methods

Statistical analyses were carried out using GraphPad Prism version 5.03 (GraphPad Software, Inc.). The tests used to evaluate the differences between groups are indicated in the legends. *p*-values < 0.05 were considered statistically significant (* *p* < 0.05; ** *p* < 0.01; *** *p* < 0.001; **** *p* < 0.0001). If not stated differently, all graphs are presented as mean of independent experiments ± standard deviation (SD).

## Figures and Tables

**Table 1:** Primary and secondary antibodies for WB and ICC.

**1st Antibody**	**Host**	**Company**	**Order No.**	**Dilution**	**WB/ICC**
Acet aTub	Mouse	Sigma-Aldrich	T7451	1:1000	WB
aSyn (Syn1)	Mouse	BD Biosciences	610786	1:1000	WB
bTubIII	Rabbit	Abcam	ab18207	1:1000	WB
bTubIII (TUJ1)	Mouse	BioLegend	801201	1.1000	WB/ICC
DDC	Rabbit	Abcam	ab3905	1:500	WB
MJFR-14-6-4-2	Rabbit	Abcam	ab209538	1:1000	Dot blot
Nestin (10C2)	Mouse	EMD Millipore Corp.	MAB5326	1:300	ICC
Sox-2 (Y-17)	Goat	Santa Cruz Biotech.	Sc-17320	1:300	ICC
Synapsin1	Mouse	Synaptic Systems	SySy106011	1:1000	WB
Tau	Rabbit	Abcam	ab64193	1:1000	WB
TH	Rabbit	EMD Millipore Corp.	AB152	1:300	WB/ICC
**2nd Antibody**	**Host**	**Company**	**Order No.**	**Dilution**	**WB/ICC**
Goat Alexa-488	Donkey	Thermo Fisher Sci.	A-11055	1:1000	ICC
Mouse Alexa-568	Donkey	Thermo Fisher Sci.	A10037	1:1000	ICC
Mouse Alexa-647	Donkey	Thermo Fisher Sci.	A-31571	1:1000	ICC
Mouse HRP	Goat	Jackson ImmunoResearch	11-035-146	1:20,000	WB
Rabbit Alexa-488	Donkey	Thermo Fisher Sci.	A21206	1:1000	ICC
Rabbit IRDye 800CW	Donkey	LI-COR Biosciences	926-32214	1:10,000	Dot blot
Rabbit HRP	Donkey	Thermo Fisher Sci.	SA1-200	1:5000	WB

## Data Availability

The data presented in this study are available in this article and the supplementary materials.

## Supplementary Materials

The following supporting information can be downloaded at: https://www.mdpi.com/article/10.3390/ijms23031812/s1.

## Abbreviations

ABC	ammonium bicarbonate
ACN	acetonitrile
aSyn	alpha synuclein
aTub	alpha tubulin
bActin	beta actin
BSA	bovine serum albumin
bTub	beta tubulin
bTubIII	beta tubulin, class III
CNS	central nervous system
CPN	cortical projection neuron
DAPI	4′,6-Diamidin-2-phenylindol
DDC	DOPA decarboxylase
EBs	embryoid bodies
FA	formic acid
hiPSCs	human induced pluripotent stem cells
ICC	immunocytochemistry
LC-MS	liquid chromatography coupled mass spectrometry
mDAN	midbrain dopaminergic neuronal cell
NPC	neural precursor cell
PD	Parkinson’s disease
PFA	paraformaldehyde
RT	room temperature
RT-PCR	reverse transcription polymerase chain reaction
*SNCA*	aSyn gene
*SNCA^Dupl^*	SNCA gene duplication
SNpc	substantia nigra pars compacta
TBS	Tris-buffered saline
TFA	trifluoroacetic acid
TH	tyrosine hydroxylase
WB	Western blot
