# Supplementary material for: Interaction of Alpha Synuclein and Microtubule Organization Is Linked to Impaired Neuritic Integrity in Parkinson’s Patient-Derived Neuronal Cells
Source: Int J Mol Sci. 2022 Feb 5;23(3):1812. doi: 10.3390/ijms23031812 (PMC8836605; doi:10.3390/ijms23031812)
Supplement: Supplementary file 1 [file ijms-23-01812-s001.zip › ijms-1554936-supplementary.pdf]

Supplementary Materials Seebauer et al.

Title: Interaction of Alpha Synuclein and Microtubule Organization Is Linked to Impaired Neuritic Integrity in Parkinson's Patient-Derived Neuronal Cells

Figure S1. Direct blue staining of total protein for dot blot analysis shown in Fig. 1A

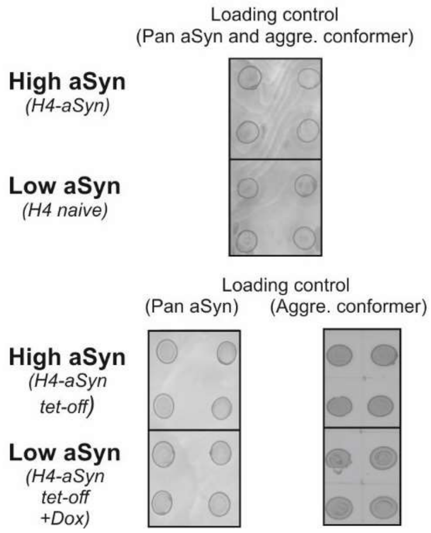

Upper panel: Direct blue staining of the dot blot membrane used for the detection of pan aSyn and aggregated conformers in H4-aSyn and H4 naïve cells shown in the upper panel of Fig. 1A.
Lower panel: Direct blue staining of dot blot membranes used for the detection of pan aSyn (left) and aggregated conformers (right) in H4-aSyn tet-off cells with or without doxycycline shown in the lower panel of Fig. 1A.

Figure S2. Ponceau staining of total protein for WB shown in Fig. 2D and F

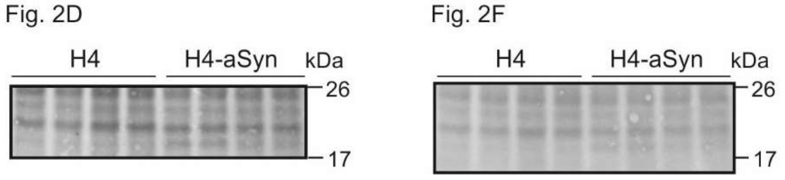

Figure S3. Generation and characterization of hiPSC-derived NPCs and mDANs

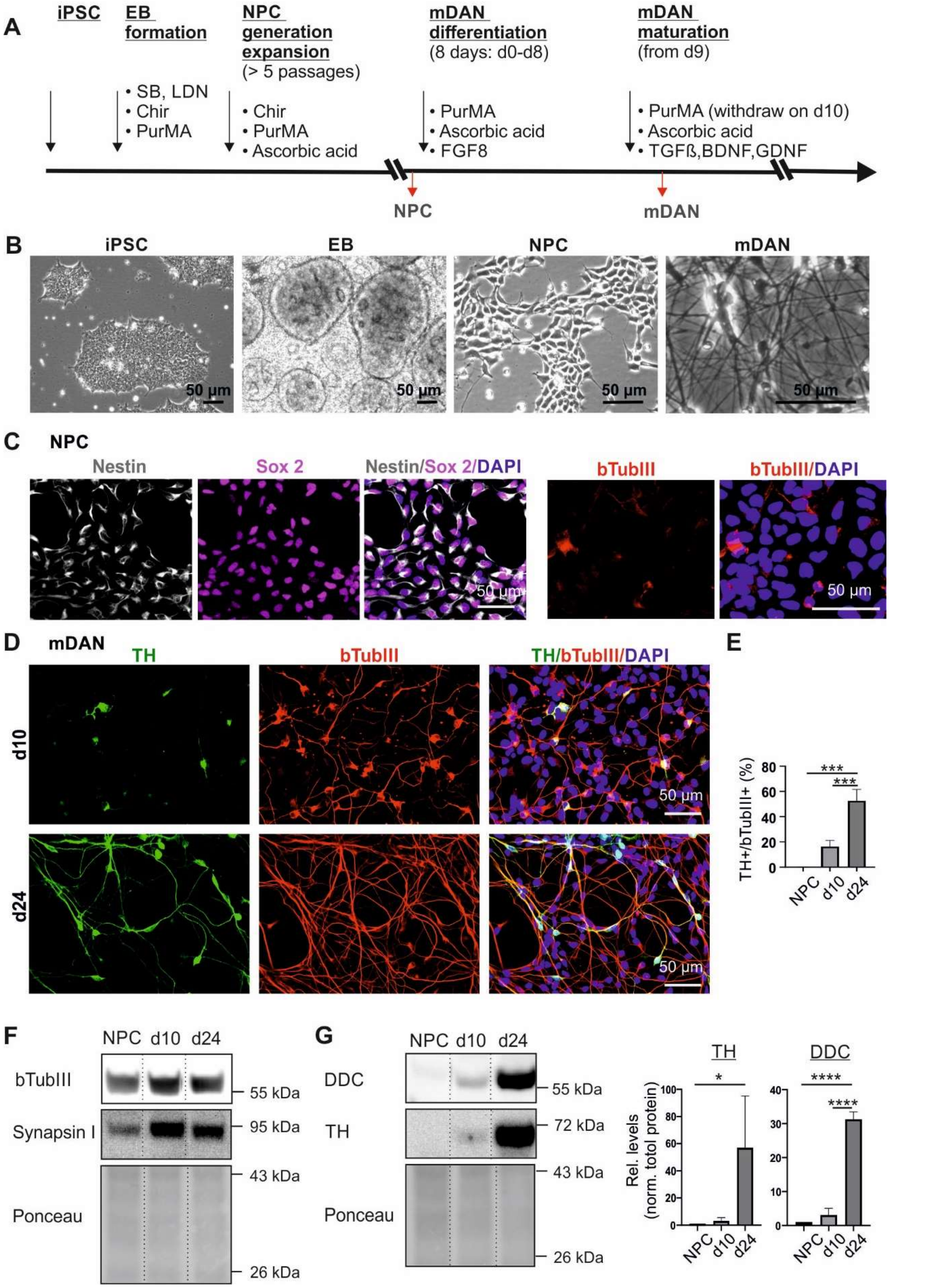

(A) Schematic summary of differentiation protocol. **(B)** Phase contrast images of hiPSCs, embryoid bodies (EBs), NPCs and mDANs. **(C)** Fluorescence images of control-derived NPCs analyzed by ICC.

NPCs coexpress the early neural precursor markers Nestin (white) and Sox2 (magenta). bTubIII is marginally expressed at the NPC stage. Cell nuclei were labelled with DAPI (blue). **(D)** Representative fluorescence images of control-derived NPCs analyzed by ICC for TH (green) and bTubIII (red) expression. Cell nuclei were labelled with DAPI (blue). **(E)** Quantification of the percentage of TH+ cells among differentiated bTubIII+ cells. TH expression was significantly upregulated after NPC differentiation toward mDANs. Statistics: One-way ANOVA followed by Holm-Sidak's multiple comparisons test. Values are shown as mean ± SD of three independent rounds of differentiation from a control hiPSC line. **(F)** Representative WB images of the neuronal markers bTubIII and synapsin I in control-derived NPCs and mDANs after maturation d10 and d24. bTubIII and synapsin I were upregulated in mDANs as compared to the levels in NPCs. **(G)** Representative WB images and the quantification of DDC and TH, the markers for dopamine synthesis, in control-derived NPCs and mDANs after differentiation for 10 and 24 days. DDC and TH expression showed a maturation-dependent increase. Lanes from different parts of the same membrane are separated by dashed lines. For all WB analyses, Ponceau staining was used for protein loading control. Relative levels were generated by normalization against Ponceau intensity and the average level of the controls and shown as mean ± SD of three independent rounds of differentiation. Statistics: One-way ANOVA followed by Tukey's multiple comparisons test. *$p < 0.05$; *** $p < 0.001$; **** $p < 0.0001$.

Figure S4. Expression analysis of cytoskeletal proteins

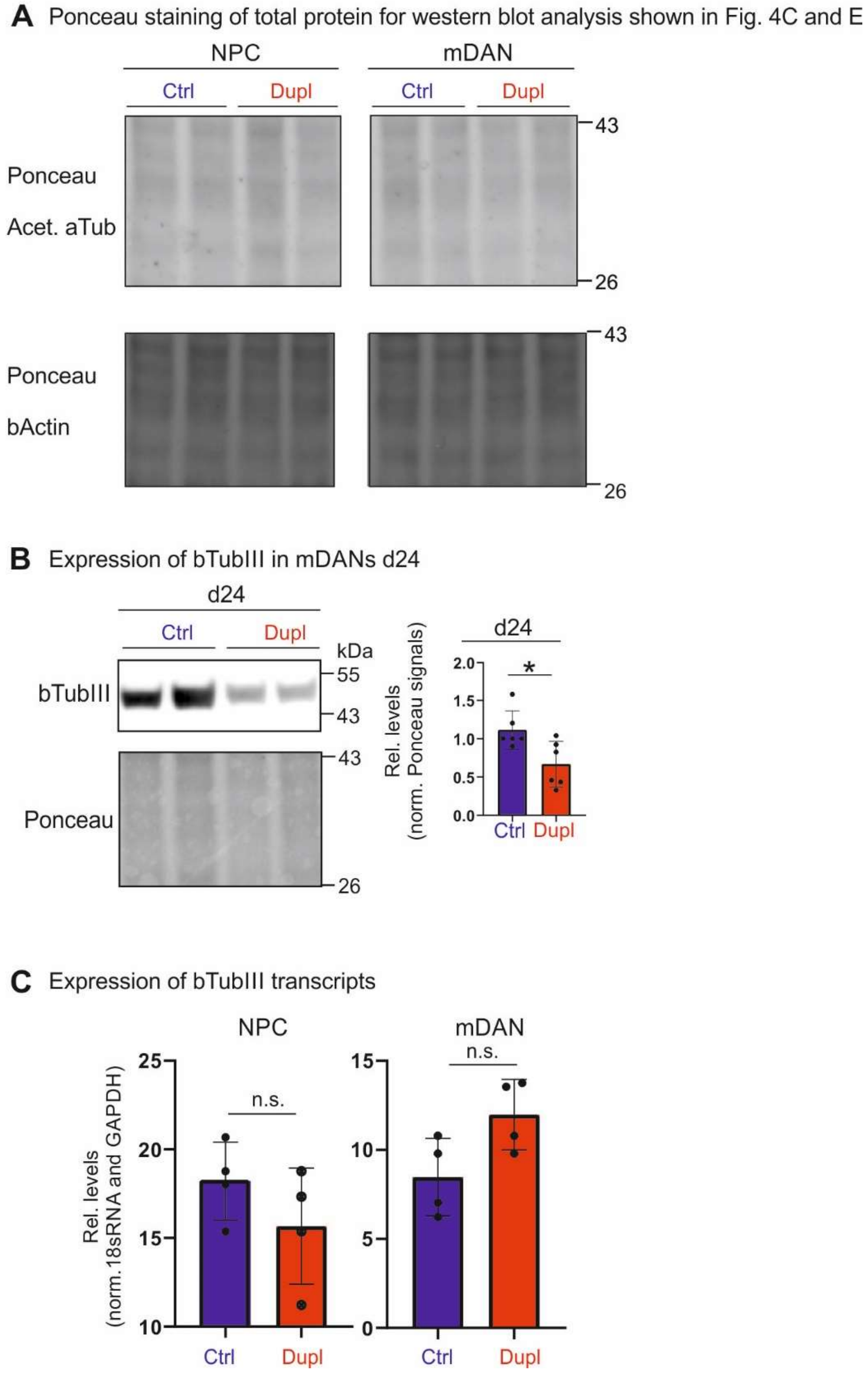

(A) Different membranes were used for detection of bTubIII, acetylated aTub and bActin shown Figure 4. These supplementary Ponceau-staining images represent the loading controls for the detection of acetylated aTub and bActin shown in Fig. 4C and Fig. 4E, respectively.

(B) Representative WB images and the quantification of bTubIII in control and *SNCA*^{Dupl} mDANs differentiated for 24 days. bTubIII levels are significantly reduced in mDANs carrying *SNCA*^{Dupl} as compared to control mDANs. Relative levels were calculated by normalization against Ponceau intensity and the level of a control line in each differentiation round. The values are shown as mean ± SD. Values from two control lines as well as two *SNCA*^{Dupl} lines and three independent differentiation rounds per cell line were used for the quantification. Statistics: Mann Whitney test; *$p < 0.05$.

(C) Quantitative reverse transcription PCR (RT-PCR) reveals unchanged transcript levels of bTubIII in *SNCA*^{Dupl} NPCs and mDANs, indicating that the reduction in bTubIII protein levels linked to *SNCA*^{Dupl} cells (Fig. 4 of the manuscript) is not regulated at the RNA level.
RNA isolation was performed by using the RNeasy® Plus MiniKit (Qiagen, Hilden, Germany) and coding DNA (cDNA) was produced via reverse transcription using the GoScriptTM Reverse Transcription System (Promega Corporation, Fitchburg, WI, USA) according to manufacturer's protocols. The cDNA concentration was adjusted to 1 ng/µL and 1 ng cDNA was applied for PCR amplification. bTubIII transcripts were amplified using specific primers (forward: ATCTTTGGTCAGAGTGGGGC; reverse: TTCATGATGCGGTCGGGATA) utilizing the LightCycler480 System (Roche, Basel, Switzerland). The amplification and detection of amplicons were performed by using the SsoAdvancedTM Universal SYBR® Green Supermix (Bio-Rad, Hercules, CA, USA). Relative quantification analysis was used to determine the relative RNA levels. bTubIII transcript levels were normalized to 18S and GAPDH levels with the following primers: 18S, forward: GGAGTATGGTTGCAAAGCTGA; reverse: ATCTGTCAATCCTGTCCGTGT; GAPDH, forward: GTCGGAGTCAACGGATTTG; reverse: TGGGTGGAATCATATTGGAAC. Gene expression levels were determined by normalization against the average of transcript levels of 18s RNA and GAPDH. Statistics: unpaired t-test.

Figure S5. Solubility analysis of aSyn in NPCs

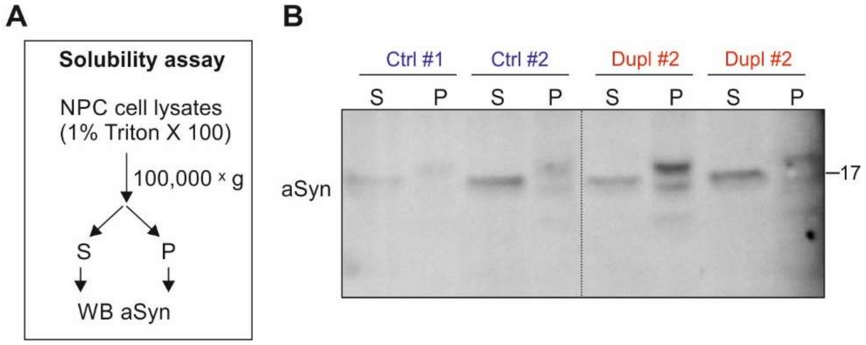

(A) Work flow of the solubility assay. NPCs were homogenized in TBS containing 1% Triton X 100. The homogenates were next centrifuged at 100,000× g for 1 h at 4°C. The pellet fraction (P) was resuspended by the equal volume of the original homogenate used for solubility assay. For WB detection of aSyn in soluble and insoluble fractions, the same volume of fractions was loaded on SDS-PAGE gels. aSyn was detected by using the antibody Syn1. **(B)** Soluble and insoluble aSyn levels in control and *SNCA*^{Dupl} NPCs are shown. aSyn in *SNCA*^{Dupl} NPCs demonstrates an increase in the insoluble fraction, characterized by aSyn immunosignals of different molecular weights. This may be attributed to increased post-translational modifications of insoluble aSyn species.
